# Daptomycin efficacy in the central nervous system of a patient with disseminated methicillin-resistant Staphylococcus aureus infection: a case report

**DOI:** 10.1186/1752-1947-6-264

**Published:** 2012-08-31

**Authors:** Fabrizio Taglietti, Floriana Campanile, Alessandro Capone, Antonino Di Caro, Elisabetta Grilli, Giulia Stazi, Taschia Bertuccio, Nicola Petrosillo, Stefania Stefani

**Affiliations:** 1 Second Infectious Diseases Division, National Institute for Infectious Diseases “LazzaroSpallanzani”, Via Portuense, 292-00149, Rome, Italy; 2 Department of Microbiology, University of Catania, Catania, Italy; 3 Department of Microbiology and Virology, National Institute for Infectious Diseases “LazzaroSpallanzani”, Rome, Italy; 4 Intensive Care Unit, National Institute for Infectious Diseases “LazzaroSpallanzani”, Rome, Italy

## Abstract

**Introduction:**

*Staphylococcus aureus* has emerged as a major nosocomial pathogen in the last decades and also represents the second most common pathogen isolated from patients in outpatient settings. Although methicillin-resistant *S.aureus* infections were traditionally limited to hospitals, community-associated cases of methicillin-resistant *S.aureus* infections have been reported. In our case, we observed an unexpected event during treatment.

**Case presentation:**

A 60-year-old Caucasian man developed fever and multiple muscle and brain abscesses caused by Panton-Valentine leukocidin-negative community-associated methicillin-resistant *S. aureus*.

**Conclusion:**

Although our patient was given antimicrobials active against the isolated methicillin-resistant *S. aureus* strain, it was only after the introduction of daptomycin that his skin, soft tissue and muscle lesions and also brain manifestations improved.

## Introduction

*Staphylococcus aureus* (SA) has emerged as a major nosocomial pathogen in the last decades and also represents the second most common pathogen isolated from patients in outpatient settings [1]. SA is commonly carried asymptomatically in human anterior nares and occasionally enters the bloodstream to cause invasive diseases with a wide range of syndromes, from minor skin and soft tissue infections to life-threatening necrotizing pneumonia and toxic shock syndrome [1]. The overall burden of staphylococcal diseases caused by antibiotic-resistant SA, particularly by methicillin-resistant *S. aureus* strains (MRSA), is increasing in several countries, including Italy, where MRSA accounts for 25% to 50% of all hospital-isolated SA strains and causes 52.8% of intensive care unit (ICU)-acquired infections [2].

Although MRSA infections were traditionally limited to hospitals, community-associated cases of MRSA (CA-MRSA) infections have been reported since the late 1990s in the USA [3]. CA-MRSA strains are genetically and phenotypically distinct from the typical multidrug-resistant healthcare-associated MRSA. These strains are resistant to beta-lactam antibiotics, are typically susceptible to other anti-staphylococcal agents, and often encode for Panton-Valentine leukocidin (PVL) and other exotoxins and virulence factors [4].

The vast majority of CA-MRSA carry one of two smaller staphylococcal cassette chromosome *mec*(SCC*mec*) types, IV and V, without the additional resistance genes, and thus are in general more susceptible to non-beta-lactam antibiotics. They appear to be associated with increased transmission and hospitalization, skin and soft tissue infection and, rarely, severe diseases including necrotizing pneumonia [5]. CA-MRSA strains have rapidly emerged worldwide and are now endemic in the USA where they are amongst the most commonly isolated pathogens in emergency departments. Furthermore, nosocomial transmission of CA-MRSA and hospital outbreaks have recently been observed in several countries [6].

Very few cases of infections due to CA-MRSA are reported in Italy [7]. We describe a case of multiple muscle and brain abscesses, caused by an MRSA encoding SCC*mec* IV, in an apparently immunocompetent patient.

## Case presentation

A 60-year-old Caucasian male retired nurse was admitted three years ago to our Infectious Diseases Hospital because of a three-day history of ingravescent dyspnoea, a skin abscess on his left arm and a very high fever. He had a past medical history of arterial hypertension, ischemic cardiomyopathy, cholecystectomy, lumbar discal hernia self-treated with steroids in case of lumbar pain, and an allergy to ceftriaxone.

Thirteen days prior to the present admission, our patient was admitted to an emergency room of another hospital for more severe lumbar pain; there he received intravenous steroids that improved his pain, and he was discharged on the same day. After three days, he consulted his general practitioner because of fever and the swelling of his left arm where a peripheral intravenous line was located during his previous emergency admission. With the suspicion of phlebitis, he was given piperacillin and calciparine for seven days. However, due to the persistence of the fever and arm swelling and the onset of dyspnoea, one week later he was admitted to a general hospital. His blood pressure was 100/60mmHg, pulse rate 120beats/min and temperature 39.5°C. Physical examination revealed right basal chest crepitations and an abscess on his left arm. Blood examinations showed a white blood cell count of 9.2 per mm^3^ with 76% neutrophils, C-reactive protein 58mg/dL (normal value <0.60mg/dL), erythrocyte sedimentation rate of 90 (normal value 0-12mm/h) and glycemia of 456mg/dL.

Red blood cells, platelet counts, and the other blood examinations were normal. A chest X-ray showed a right basal consolidation. On the second day of admission, his skin abscess was surgically drained, yielding *S. aureus* with a vancomycin minimum inhibitory concentration of 1μg/mL. On the same day, our patient developed chest pain and was admitted to the cardiologic ICU for an inferior ST segment elevation myocardial infarction.

**Figure 1:**
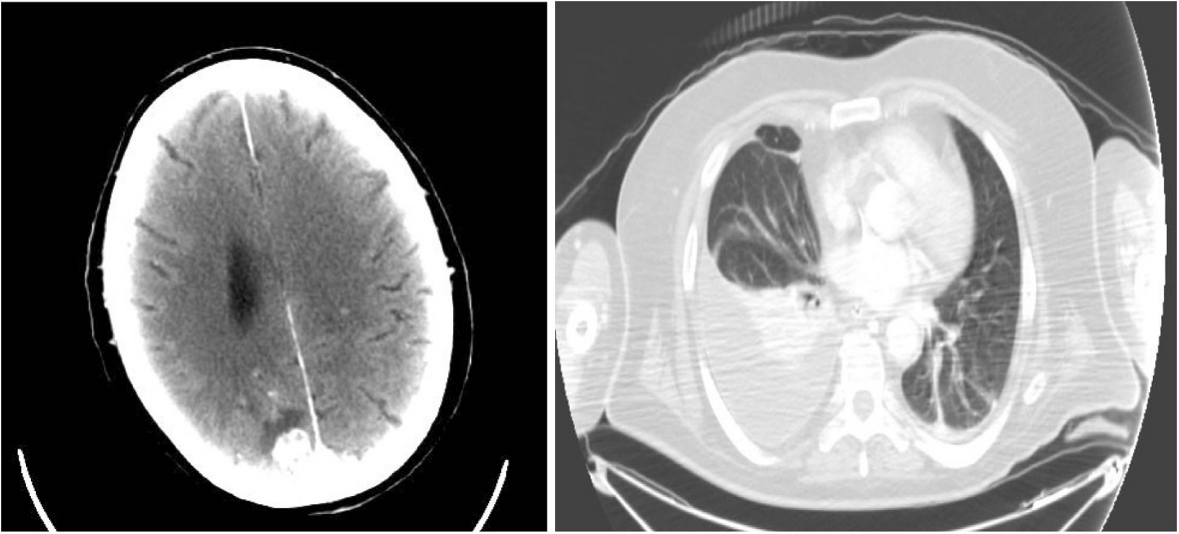
Computed tomography of the brain and thorax.

On day 4, his clinical conditions worsened and our patient presented with seizures: total-body computerized tomography showed multiple cerebral lesions described as brain abscesses, right pneumonia and scapular soft tissue abscesses (Figure 1). Blood cultures were then performed. He was started on imipenem (500mg intravenously three times a day), ciprofloxacin (200mg twice daily) and teicoplanin (200mg once daily).

On day 5, because of persisting fever and dyspnoea, our patient was transferred to the ICU of our Infectious Disease Institute. At the moment of ICU admission, he was febrile (39.8°C), confused and dyspnoic: blood and draining cultures were performed, and empiric antibiotic therapy, including meropenem (1g intravenously three times per day), linezolid (600mg intravenously twice daily) and levofloxacin (500mg intravenously twice daily), was started. Urinary antigen tests for *Legionella* and *Streptococcus pneumoniae* were negative.

His fever still persisted by day 6. A vertebral nuclear magnetic resonance (NMR) imaging study was negative for spondylodiscitis and transthoracic echocardiography showed no endocardial vegetations. On day 7, another surgical drainage of his left arm and scapular soft tissue abscesses was performed. His clinical condition was stable. Both the blood cultures and swabs performed in our Infectious Diseases Hospital yielded MRSA.

Both strains of SA isolates were methicillin-resistant. Molecular characterization was conducted by polymerase chain reaction of *mec*A, *pvl* and major enterotoxin content genes, SCC*mec*-typing and multilocus sequence typing (MLST); pulsed-field gel electrophoresis (PFGE) was used only to define possible relationships among the isolates. All techniques were performed as previously described [8,9]. Strains were identical, as demonstrated by PFGE (data not shown) and belonged to the ST8 SCC*mec* IV.3. A further characterization to define the virulence gene content showed that the strains were PVL-negative, but possessed different superantigen toxins (enterotoxins k, q, d, j and tst), all conferring the hypervirulent behavior demonstrated by the strains. The isolated strains were susceptible to rifampin, fluoroquinolones, gentamicin, tetracycline and cotrimoxazole and were also fully susceptible to glycopeptides, linezolid, daptomycin, quinupristin/dalfopristin and tigecycline. The SA strain isolated during the previous admission to another hospital was not available to perform further microbiologic analysis.

On day 12, a whole-body computed tomography showed that the multiple brain abscesses previously described had worsened, and new brain lesions were evident. In addition, computed tomography showed the presence of left basal pneumonia and the onset of new multiple muscle abscesses (left deltoid, paravertebral, left pectoral and right psoas, gluteal and iliac). A neurosurgery consultation excluded the possibility of draining the brain abscesses. Daily administration of daptomycin 6mg/kg was started and the administration of meropenem was interrupted.

On day 22, the clinical and radiologic findings improved. Our patient became non-febrile, and erythrocyte sedimentation rate and C-reactive protein decreased. On day 38, a chest X-ray was normal, daptomycin and levofloxacin were interrupted, and our patient was discharged in good general condition. Administration of linezolid 600mg twice a day was continued for two weeks. After a two-year period, our patient is still in good health.

## Discussion

Skin and soft tissue and lower respiratory infections account for much of the current clinical literature about CA-MRSA. We report the case of a patient with pneumonia and multiple abscesses involving skin, soft tissue and the central nervous system caused by a PVL-negative CA-MRSA.

Notably, in our patient we observed a rapid onset and dissemination of the infection and clinical severity, similar to cases of PVL-positive CA-MRSA infections already described in the literature. Community-isolated strains, not only the PVL-positive ones, can be considered one of the most significant challenges of this epidemiological change of SA. In fact, these CA-MRSA strains, even if PVL negative, possess several virulence determinants responsible for more severe diseases than the nosocomial clones. One of these virulence determinants of CA-MRSA strains is represented by the presence of staphylococcal superantigens that, as described in the literature, may be related to steroid therapy [8]. Our patient, as seen in the case report, had a history of lumbar discal herniation self-treated with steroids and the MRSA strain isolated possessed several superantigen toxins.

Although our patient was given antimicrobials that were active against the isolated MRSA strain, namely levofloxacin and linezolid, his clinical condition rapidly worsened. A likely explanation is the bacteriostatic effect of linezolid, the concentration-dependent activity of levofloxacin [9] and an aggressive virulence of the MRSA strain. In our case, the introduction of daptomycin in the antimicrobial schedule allowed fast improvement concerning skin, soft tissue and muscle lesions and also brain manifestations. Indeed, daptomycin has been shown to be effective in central nervous system infection in humans and animal models [10,11].

## Conclusions

CA-MRSA infection, even if PVL negative, can be rapidly aggressive with multiple lesions, including in the brain. Daptomycin seems to be effective in the treatment of these severe cases owing to its pharmacokinetic and pharmacodynamic characteristics and its fast bactericidal activity with a limited bacterial lysis [12,13].

## Consent

Written informed consent was obtained from the patient for publication of this manuscript and accompanying images. A copy of the written consent is available for review by the Editor-in-Chief of this journal.

## Competing interests

The authors declare that they have no competing interests.

## Authors’ contributions

FT and GS analyzed and interpreted the patient data regarding the infectious disease. FC, SS, ADC and TB analyzed microbiological data. AC and EG collected the patient’s clinical data. NP was a major contributor in writing the manuscript. All authors read and approved the final manuscript.

## References

[B1] Lowy FD Staphylococcus aureus in fections N Eng J Med 1998 339 520 532 10.1056/NEJM199808203390806 9709046

[B2] Malacarne P Boccalatte D Acquarolo A Agostini F Anghileri A Giardino M Giudici D Langer M Livigni S Nascimben E Rossi C Bertolini G Epidemiology of nosocomial infection in 125 Italian intensive care units Minerva Anestesiol 2010 76 1 13 23 20125069

[B3] Moran GJ Krishnadasan A Gorwitz RJ Fosheim GE McDougal LK Carey RB Talan DA EMERGEncy ID Net Study Group Methicillin resistant Staphylococcus aureus infections among patients in the emergency department N Engl J Med 2006 355 666 674 10.1056/NEJMoa055356 16914702

[B4] Yeung M Balma-Mena A Shear N Simor A Pope E Walsh S McGavin MJ Identification of major clonal complexes and toxin producing strains among Staphylococcus aureus associated with atopic dermatitis Microbes Infect 2011 13 2 189 197 10.1016/j.micinf.2010.10.023 21093604

[B5] Francis JS Doherty MC Lopatin U Johnston CP Sinha G Ross T Cai M Hansel NN Perl T Ticehurst JR Carroll K Thomas DL Nuermberger E Bartlett JG Severe community-onset pneumonia in healthy adults caused by methicillin-resistant Staphylococcus aureus carrying the Panton-Valentine leukocidin genes Clin Infect Dis 2005 40 100 107 10.1086/427148 15614698

[B6] Otter JA French GL Nosocomial transmission of community-associated methicillin resistant Staphylococcus aureus: an emerging threat Lancet Infect Dis 2006 6 753 755 10.1016/S1473-3099(06)70636-3 17123892

[B7] Stefani S Bongiorno D Cafiso V Campanile F Crapis M Cristini F Sartor A Scarparo C Spina D Viale P Pathotype and susceptibility profile of a community-acquired methicillin-resistant Staphylococcus aureus strain responsible for a case of severe pneumonia Diagn Micr Infec Dis 2009 63 100 104 10.1016/j.diagmicrobio.2008.09.012 19073304

[B8] Schlievert PM Case LC Strandberg KL Abrams BB Leung DY Superantigen profile of Staphylococcus aureus isolates from patients with steroid-resistant atopic dermatitis Clin Infect Dis 2008 46 1562 1567 10.1086/586746 18419342 PMC2637450

[B9] Bolon MK The newer fluoroquinolones Infect Dis Clin N Am 2009 23 1027 1051 10.1016/j.idc.2009.06.003 19909896

[B10] Gerber P Stucki A Acosta F Cottagnoud M Cottagnoud P Daptomycin is more efficacious than vancomycin against methicillin-susceptible Staphylococcus aureus in experimental meningitis J Antimicrob Chemoth 2006 57 720 723 10.1093/jac/dkl007 16459345

[B11] Le J Bookstaver PB Rudisill CN Hashem MG Iqbal R James CL Sakoulas G Treatment of meningitis caused by vancomycin-resistant Enterococcus faecium: high-dose and combination daptomycin therapy Ann Pharmacother 2010 44 12 2001 2006 10.1345/aph.1P333 21119097

[B12] Cotroneo N Harris R Perlmutter N Beveridge T Silverman JA Daptomycin exerts bactericidal activity without lysis of Staphylococcus aureus Antimicrob Agents Chemother 2008 52 6 2223 2225 10.1128/AAC.01410-07 18378708 PMC2415783

[B13] Utili R Cogo A Cristini F Prisco V Sagnelli E Tascini C Iacoboni C Capone A Gattuso G Angarano G Petrelli E Grossi P Bartezaghi M Zagni E Clinical experience with daptomycin in Italy: results from a registry study of the treatment of Gram-positive infections between 2006 and 2009 J Chemother 2012 24 2 113 121 10.1179/1120009X12Z.00000000023 22546768

